# Detection of hydrogen using graphene

**DOI:** 10.1186/1556-276X-7-198

**Published:** 2012-03-23

**Authors:** Robert C Ehemann, Predrag S Krstić, Jonny Dadras, Paul RC Kent, Jacek Jakowski

**Affiliations:** 1 Department of Physics and Astronomy, Middle Tennessee State University, Murfreesboro, TN, 37130, USA; 2 Physics Division, Oak Ridge National Laboratory, Oak Ridge, TN, 37831, USA; 3 Department of Physics and Astronomy, University of Tennessee, Knoxville, TN, 37996, USA; 4 Center for Nanophase Materials Sciences, Oak Ridge National Laboratory, Oak Ridge, TN, 37831, USA; 5 National Institute of Computational Sciences, University of Tennessee, Oak Ridge, TN, 37831, USA

**Keywords:** Graphene, DFTB, Hydrogen detection, HOMO-LUMO gap, Molecular dynamics

## Abstract

Irradiation dynamics of a single graphene sheet bombarded by hydrogen atoms is studied in the incident energy range of 0.1 to 200 eV. Results for reflection, transmission, and adsorption probabilities, as well as effects of a single adsorbed atom to the electronic properties of graphene, are obtained by the quantum-classical Monte Carlo molecular dynamics within a self-consistent-charge-density functional tight binding formalism We compare these results with those, distinctly different, obtained by the classical molecular dynamics.

PACS: 61.80.Az, 61.48.Gh, 61.80.Jh, 34.50.Dy.

## Background

The sp^2 ^hybridized carbon allotrope, graphene, has recently shown particular promise in applications such as nanoscale electronics, hydrogen storage [1], and nanosensors. This is due to the material's outstanding thermal and electronic properties. The sensitivity of the electronic properties of a single graphene sheet to small defects in its 2-D crystal structure and chemical composition indicates a possibility of its application as a few-particle detector [2-4]. Graphene-based electronics in space vehicles might also be sensitive to the damages caused by cosmic radiation containing a wide spectrum of particles, a significant component of which would be light atoms from the solar wind. The significance of studies of graphene bombarded by hydrogenic atoms in understanding the damages of the CFC carbon tiles in the divertor of a fusion reactor (ITER) to the plasma irradiation has also been stressed recently [5,6]. These defects include lattice defects, with possible creation of vacancies, as well as chemical changes induced by the hydrogen sticking to the lattice [7,8]. The resultant changes in the electronic conductance due to changes in the electronic structure have also been studied [3,9]. For example, work by Deretzis et al. [2] has shown that even single vacancy deformations in graphene nanoribbons can have measurable effects on the material's conduction properties. These applications all motivate our study of energetic particle impact with graphene.

In this paper, we study the perpendicular impact of hydrogen on a single graphene sheet over more than three decades of impact energies (0.1 to 200 eV) using methods of quantum-classical Monte Carlo molecular dynamics. Our approach is described in detail in the second section entitled 'Methods'. The irradiated target was an infinite graphene sheet obtained by applying 2-D periodic boundary conditions to a graphene cell of size 29.12 × 28.53 Å (336 C atoms). The graphene was prepared at a temperature of 300 K by a Nose-Hoover thermostat and left free during each collision event, which lasted 200 to 500 fs, depending on the impact energy. The irradiation was performed by more than 1,000 independent trajectories for each impact energy, with randomly chosen position of emission of an atom above the surface of the graphene cell. In this method, the total electronic energy of the system is solved quantum-mechanically at the beginning of each time step (on the order of a femtosecond), maintaining fixed atom positions; after incorporating the nucleus-nucleus interaction into the total electronic energy, forces on each atom are updated, and the atoms are moved classically within the time step. The electronic structure is solved here by the self-consistent-charge-density functional tight binding (SCC-DFTB) method [10-12]. To allow for the high-energy impact, we fit the original SCC-DFTB parameters [13] at close distances (< 0.2 Å) to the binary Ziegler-Biersack-Littmark (ZBL) [14] repulsive potentials.

Results for reflection and transmission probabilities, angular distributions, and adsorption probabilities at low energies (0.1 to 1 eV) are shown and analyzed in the first part of the 'Results and discussion' section, entitled 'Irradiation dynamics and effects on electronic structure'. Additionally, changes in the molecular orbital levels close to the Fermi energy, which influence the non-equilibrium ballistic electron transport properties (i.e., the electric conductance) of the system, are calculated and characterized by the changes, ∆*E_l-h_*, in the difference, *E_l-h_*, of the (discrete) lowest unoccupied molecular orbital and highest occupied molecular orbital energies in response to the hydrogen adsorption. These changes are indicative of possible changes in the graphene sheet conductance. They are, surprisingly, on the order of 1 eV and depend on the vibrational energy of the adsorbed hydrogen. Adsorption occurs only for the low-energy impacts (< 1 eV). This confirms some predictions in literature on the extreme sensitivity of the highest occupied molecular orbital (HOMO)-lowest unoccupied molecular orbital (LUMO) gap and transport properties of graphene and SWCNT to the adsorption of hydrogen and other atoms and molecules [15-19].

In the second part of the 'Results and discussion' section entitled 'Comparison with classical molecular dynamics', we perform classical molecular dynamics (CMD) calculations with two state-of-the-art bond order hydrocarbon potentials, reactive empirical bond-order (REBO) [20] and adaptive intermolecular reactive empirical bond order (AIREBO) [21]. We use the corrected set of the classical potentials [22] to allow high impact energies and compare the classical MD probabilities with our quantum-classical results. Although CMD with these potentials is significantly faster than SCC-DFTB, allowing for longer timescales, larger systems, and greater energy ranges to be studied, it turns out that the classical potentials are of limited applicability for the studied system and dynamics. We hope that this data motivates improvements to these potentials since their speed is very attractive for radiation damage-type problems. Our conclusions are given in the final section.

## Methods

To simulate effects of irradiation on graphene, one can apply direct molecular dynamics methods in which electronic structure is treated explicitly using quantum mechanics, while the motion of the nuclei is described by the means of the classical dynamics. This allows one to accurately describe bond breaking and formation as well as the interatomic potentials. Such an approach is, however, computationally very expensive, which greatly limits the system sizes, timescales, and choice of quantum mechanics-based methods. To mimic the dynamics observed by experiment, we apply a Monte Carlo approach to the trajectories, i.e., using a large number of trajectories, randomly varying 'impact parameters' to obtain acceptable statistics of the collision events. Even using this approach, we must use a less expensive and more approximate quantum-mechanical approach. Here, we use the SCC-DFTB method, an approximate density functional theory (DFT) method in which only valence electron interactions are considered. Although a full DFT treatment would be ideal, this is currently too expensive computationally, even for a handful of trajectories. In SCC-DFTB, the total electronic densities and energies are expressed by solution of the Schrodinger equation in the Kohn-Sham form, using predetermined Hamiltonian and overlap integrals as well as repulsive splines fit to reference systems (so-called Slater-Koster parameters). The tight binding methods applied to the large (solid-state) systems have a long history. Here, we use a self-consistent charge version developed by Bremen Group (Bremen, Germany) [10-12]. SCC is a second-order correction term in the DFTB total energy involving interactions between localized fluctuations of the electron density; it uses an iterative procedure to converge on the new electron density at each time step. In this SCC-DFTB method, spin polarization is neglected. We employed a Fermi-Dirac smearing with electronic temperature *T*_el _= 1,000 K, which has a similar effect to averaging over many electronic states near the Fermi level.

**Figure 1:**
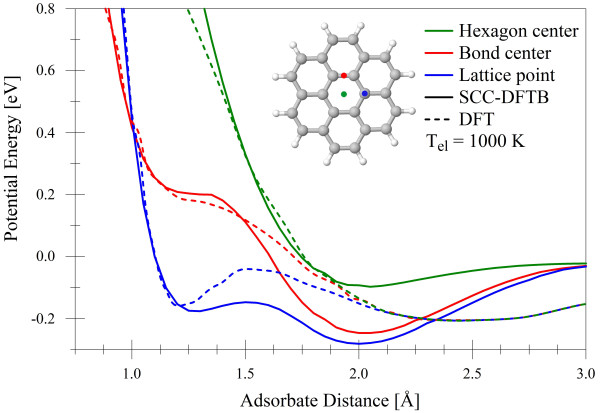
**SCC-DFTB and LDA-DFT **[25]**potential energies of the hydrogen-coronene interaction**.

To safely allow for high-energy bombardment simulations (in our case 200 eV), we use a refitted version of the original DFTB PBC-0-3 [13] parameters obtained by fitting to the ZBL [14] repulsive interactions at short distances (< 0.2 Å). The PBC-0-3 parameters used here have already shown good results for the hydrogenation of periodic graphene [23] at thermal energies. We show in Figure 1 the potential energy curves of a hydrogen atom interacting with a coronene molecule obtained by the SCC-DFTB using PBC-0-3/ZBL parameters and by DFT using a local density approximation functional [24]. At distances closer than 1.5 Å, agreement between DFT [25] and DFTB potentials is quite good. Between 1.5 and 4 Å, DFTB potentials overestimate bond strength, and wells are about 0.5 Å closer to the surface than their DFTB counterparts. Also notable is the lack of convergence of the three potentials until they approach 0 eV. Although SCC-DFTB underestimates bonding at the bond center and lattice point positions, these are qualitatively similar to DFT potentials [25]. The problem of thermal atom adsorption gave rise to many experimental and theoretical papers [7,15-19] and references therein. The previously reported SCC-DFTB studies [26] of collision-induced reactions in carbon materials within the same energy range considered here were in excellent agreement with experimental findings. Additional comparisons between DFT and SCC-DFTB are contained in the studies of Zheng et al. and Elstner [27-29].

**Figure 2:**
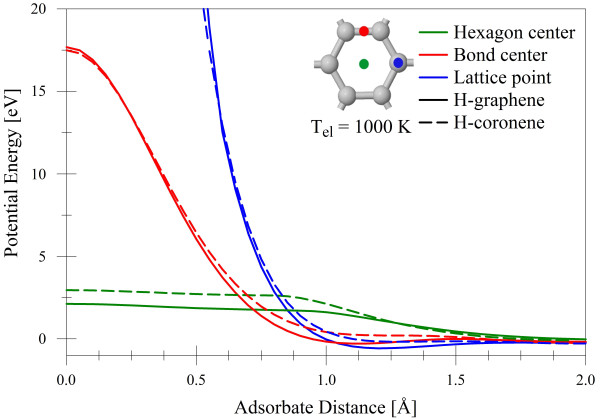
**Potential energy of the H-graphene and H-coronene interactions at analogous points in the lattice**. As calculated by the SCC-DFTB with the PBC-0-3/ZBL parameters.

Figure 2 compares the SCC-DFTB potential energy of the hydrogen-graphene and hydrogen-coronene interactions as a function of *z*-position above the graphene/coronene plane. The coronene potentials show bonding that is roughly 1 eV weaker and a potential barrier at the hexagon center that is 1 eV higher, reflecting the changes in electronic structure between hydrogen-terminated and periodic sp^2 ^carbon. Despite these differences, the forms of the H-graphene and H-coronene interactions are very similar. Thus, the agreement of SCC-DFTB with DFT calculations of the coronene molecule in Figure 1 indicates that the PBC-0-3/ZBL SCC-DFTB parameters are as acceptable for use with graphene as the DFT approach.

Notably, there are two bonding regions in the H-graphene potential. For incidence directly upon a lattice carbon, the potential minimum occurs at approximately 1.1 Å, while incidence upon a C-C bond center shows a shallower potential with minimum close to 1.0 Å. Indeed, there are many potential wells in the 3-D multibody potential that are responsible for capturing impinging hydrogen atoms; these will later be shown to have an effect on the electronic structure of resultant H-graphene surfaces. There are repulsive barriers at the bond center and hexagon center of heights 17 and 2.5 eV, respectively. Notably, hydrogen encounters no barrier before entering the potential well when incident directly on a lattice carbon.

About ten per decade incident kinetic energies ranging from 0.1 to 200 eV are considered for the impinging hydrogen atom. While cumulative bombardment is not investigated, 1,008 single impact simulations are performed for each incident energy; this is achieved using 1,008 processors, one for each trajectory, on the Kraken Cray XT5 supercomputer (National Institute of Computational Sciences, University of Tennessee, Knoxville, TN, USA). The target graphene surface described in the 'Background' is situated in the *z *= 0 plane and periodically extended in the *xy *coordinate plane. To simulate the bombardment in a real-world environment, the sample is thermostated (via Nose-Hoover scheme) to 300 K before bombardment and left to evolve freely during approximately 0.1 to 1 ps (depending on incident energy) simulation time. The impinging hydrogen atom is released from a random (*x*, *y*) position in the *z *= 10 Å plane, with velocity perpendicular to the graphene sheet.

## Results and discussion

### Irradiation dynamics and effects on electronic structure

**Figure 3:**
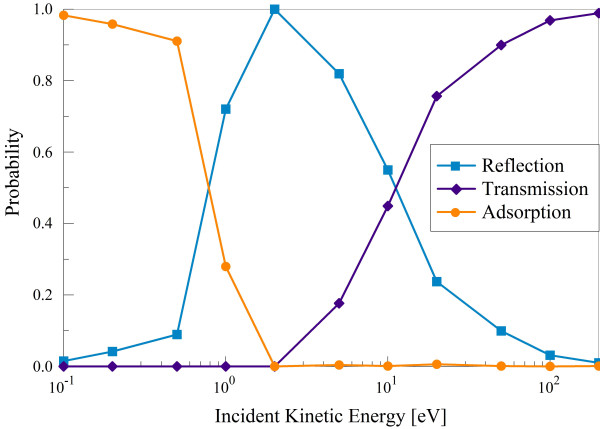
**Probabilities of reflection, transmission, and adsorption as a function of incident kinetic energy**.

Three outcomes of the bombardment are observed: reflection, transmission, and adsorption of the incident hydrogen atom; no sputtering of any type was observed in our quantum-classical approach. Figure 3 shows the probabilities of these processes as a function of incident H-atom energy. At 20 eV and above, transmission is the dominant process, as expected from the potentials in Figure 2. At the midrange energies of 1 to 10 eV, reflection is primarily observed, with a peak at 2 eV. At 1 eV, H still transfers enough kinetic energy to the target carbon atoms to allow its bonding in the wells of depth approximately 0.5 eV near the lattice points and bond centers. As the incident energy becomes comparable with the depth of this and smaller wells, adsorption becomes the dominant process as expected.

**Figure 4:**
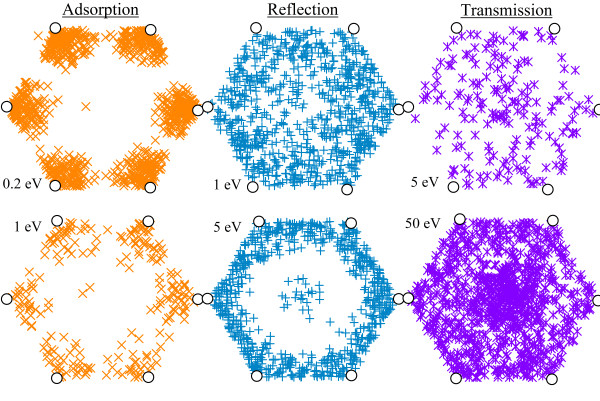
**Positions of reflection, transmission, and adsorption events for the quantum-classical calculations**. In a representative graphene hexagon, using SCC-DFTB. Adsorption (left) shows clustering of hydrogen atoms around the lattice carbons. Reflection (center) is most probable at the perimeter of the hexagon where interactions are strongest. Transmission (right) can occur at most points in the lattice for high energies but tends to occur at the hexagon center due to the low barrier.

Reflection of the incident hydrogen can occur at all points in the graphene lattice. As can be seen in Figure 2, the threshold for transmission is approximately 2.5 eV at the hexagon center. These atoms are still of insufficient energy to penetrate the barrier at the C-C bond position, so those that do not impact near the center of the hexagon are reflected (see Figure 4).

By examining the position within the hexagon where incident atoms are reflected, transmitted, or adsorbed, one can infer the form of the many-body potential at nonsymmetrical parts of the lattice. Figure 4 shows the hexagon-localized reflection, transmission, and adsorption for several energies. Lattice positions represented in Figure 4 are the turning points for reflection, closest approach positions for transmission, and final *x*-*y *positions for adsorption. Adsorbed atoms are clustered around the carbon atoms, often showing some lateral vibration.

Reflection is distributed evenly around the perimeter of the hexagon, indicating that incident atoms are deflected away from the hexagon center due to the relatively low force experienced here. Also due to the weak interaction at the hexagon center, it is the most probable location for transmission to occur. Thus, atoms incident upon or deflected toward this position are both able to penetrate. These results agree with those from a previous study [30], which found that reflection occurs at all points in the hexagon, and transmission is most probable near the hexagon center.

**Figure 5:**
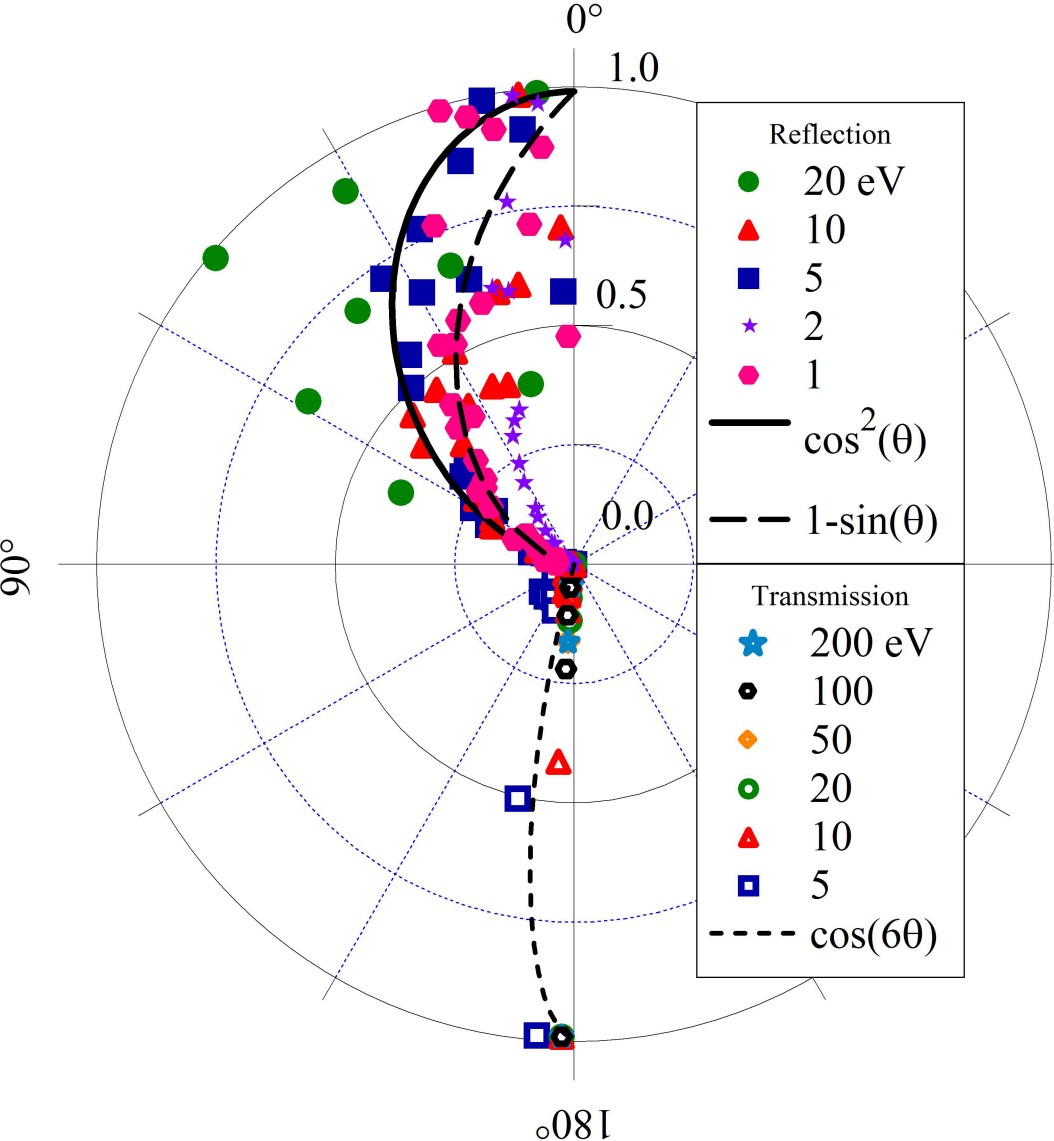
**Angular distributions of reflected (*θ *< 90°) and transmitted (*θ *> 90°) hydrogen atoms**. Distributions found to fit the data are shown in black.

The scattering of incident noble gas atoms has been investigated at high energies (keV), where transmitted particles were found to have very little angular deflection while leaving the graphene relatively unaffected [8]. Here, similar results are found for hydrogen at lower energies. Figure 5 shows the angular cross sections of reflection and transmission for our SCC-DFTB results. Radii are normalized to unity for the purpose of comparison. Cross sections are calculated according to

(1) $$\frac{dN}{d\Omega }≅\frac{1}{2\pi \;N_{\text{max}}}\frac{N\left(\theta \pm \Delta \theta /2\right)}{\text{sin}\theta \;\Delta \theta }$$

Here, 1/*N*_max _normalizes the distribution, and the differential solid angle *d*Ω becomes 2π sin *θ dθ *due to the azimultal symmetry of the problem. *N*(*θ *± Δ*θ*/2) is the number of atoms scattered into a bin of width Δ*θ *centered at polar angle *θ*.

Small changes in the *x*- or *y*-components of an atom's linear momentum are much more visible for low incident energies, where these changes can be comparable to the initial momentum. In the SCC-DFTB simulations, atoms with such low incident energy tend to reflect when not adsorbed, and the reflected angular distribution shows much more scattering. Transmitting hydrogen atoms in these simulations tend to have higher incident energies, so the small *x*- or *y*-forces don't produce a significant angular displacement of their momenta. While atoms incident at 5 and 10 eV have a wider distribution than at the higher energies, they tend to penetrate only near the center, where the H-C interactions are weakest.

The dominance of adsorption in SCC-DFTB simulations at impact energies below 1 eV provides enough statistical weight for an investigation of the effects of H-adsorption on the *E_l-h _*quantity of the affected graphene. However, roughly a third of the incident atoms are found to bond to the surface after initially being reflected at a large angle relative to their initial momenta. These 'wandering' hydrogen atoms, primarily seen at 0.5 eV incidence, generally drift above the graphene surface at a distance of about 3 Å for 2 to 5 fs before falling toward a lattice carbon and adsorbing. Roughly 10% of these 'wanderers' do not bond to a carbon within the simulation time. Therefore, while they are counted as adsorbed in Figure 3, they are ignored in the henceforth analysis to reduce uncertainties.

The graphene band gap is often computed using a band structure or density of states calculation. However, the graphene system studied here is subject to thermal motion as well as bombardment, and the impinging particle should not be included in Brillouin zone integration. As discussed earlier, we simply define a quantity *E_l-h _*by subtracting the energy of the highest occupied orbital from that of the lowest unoccupied orbital. The 1,000-K electronic temperature used creates a 'smearing' of the orbital occupations near the Fermi level. We use occupations of 1.8 for *h *(analogous to the HOMO) and 0.2 for *l *(analogous to the LUMO). This allows us to accomplish significant statistics while accounting for the different sites of adsorption and variety of vibrational states in which atom is adsorbed. The system is a 336-atom supercell, equivalent to an 18 × 18 × 1 k-point grid.

**Figure 6:**
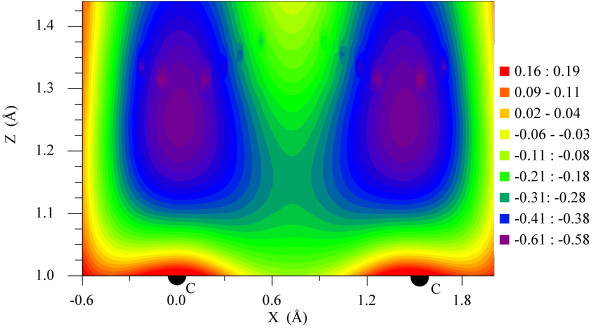
**A contour plot of the potential energy of a H-atom**. In vicinity of the two adjacent carbon bonding centers (C) in graphene, Z being the direction orthogonal to the graphene. The depths of the wells in which hydrogen bonds are equal.

**Figure 7:**
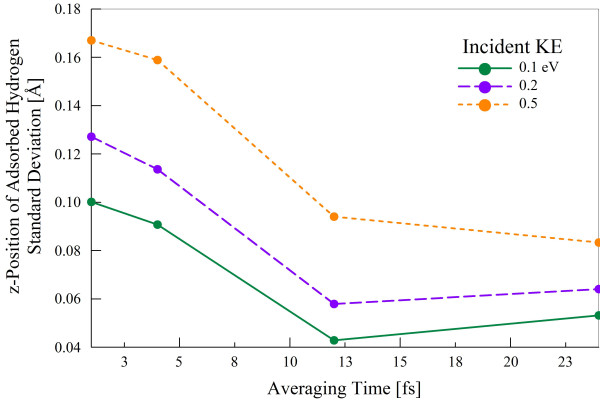
**Standard deviation of hydrogen *z*-position distribution as a function of averaging time**.

Figure 6 shows contour plots of the two equivalent potential wells for hydrogen, corresponding to two adjacent C atoms of graphene. The depth of the wells is about -0.61 eV. Thus, when the kinetic energy of H is comparable to the well depth, excited vibrational motion is possible after adsorption; to account for this, we average the change in *E_l-h _*over a number of time steps at the end of the simulation. When doing this time-averaging, it is important to avoid time steps at which some of the hydrogen atoms have not yet bound to the graphene surface. Figure 7 displays the standard deviation of the hydrogen *z*-position distribution averaged over 1, 4, 12, and 24 fs of simulation time. In all cases, the mean value is within a single standard deviation of 1.2 Å.

One can see that the standard deviation, representative of the distribution's width, is higher for low averaging times. Additionally, atoms incident with higher kinetic energy are adsorbed with greater vibrational energy, so they display a wider distribution of *z*-positions. As shown in Figure 7, the wider distributions that come with this higher vibrational energy produce a smaller change in the *E_l-h _*on average.

**Figure 8:**
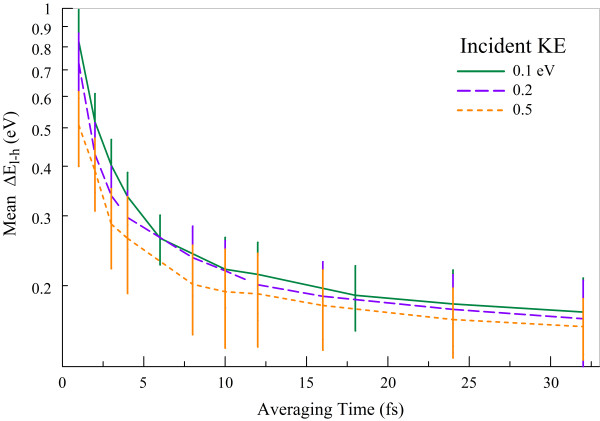
**Mean change in *E_l-h _*as a function of averaging time for three incident kinetic energies**.

Previous studies [31,32] have found that a tuning of the graphene band structure can be achieved by partial or full hydrogenation of nanoribbons, achieving band gaps of 0.43 to approximately 4.0 eV. The results obtained here support this, showing a sensitivity of the graphene band gap to even a single stuck hydrogen atom. At the largest averaging time considered here, the average change in *E_l-h _*is 171.5 meV for 0.1 eV incidence, 165.1 meV for 0.2 eV incidence, and 157.7 meV for 0.5 eV incidence (Figure 8). As discussed above, *E_l-h _*is not equal to the band gap, though it is correlated with it. There is a nonlinear relationship between the change in *E_l-h _*and *z*-position of hydrogen, which is the source of the difference between these results.

**Figure 9:**
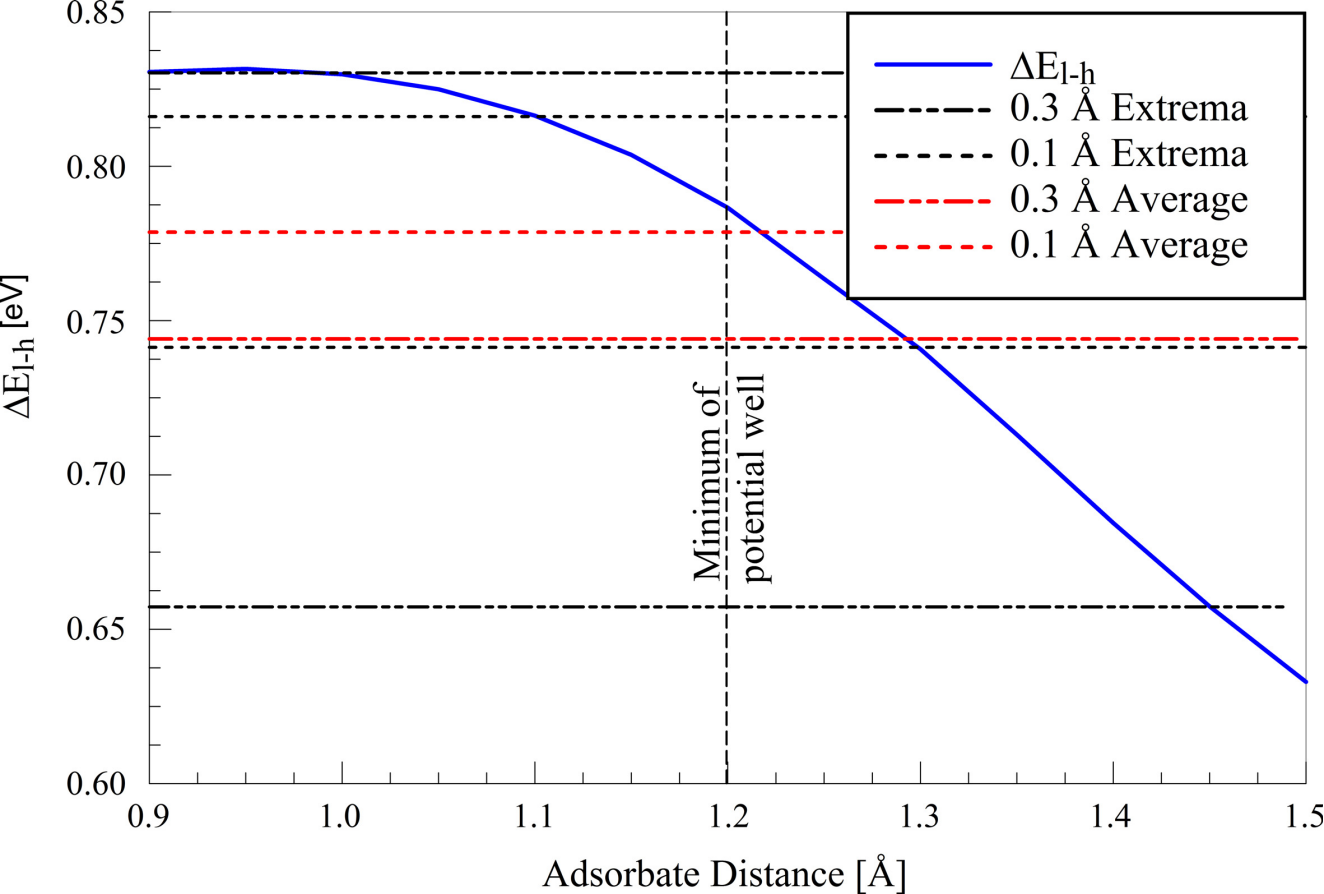
**Mean change in *E_l-h _*as a function of adsorbate hydrogen distance**. Displayed are maximum, minimum, and average changes for typical large and small oscillation amplitudes resulting from 0.5 and 0.1 eV bombardments, respectively. Calculations are performed using an ideal graphene plane.

Figure 9 shows the change in the *E_l-h _*quantity as a function of adsorbate distance for an ideal graphene plane. Since the hydrogen is directly above a lattice carbon, the minimum of the potential well is located at 1.2 Å. The average minima and maxima of low-energy (0.1 eV incidence) and high-energy (0.5 eV incidence) oscillations are 0.1 and 0.3 Å, respectively. The change in *E_l-h _*decreases with increasing adsorbate distance, and the flattening observed below 1 Å causes the minimum position in oscillation to affect the gap less than the maximum position. Thus, larger oscillation amplitudes produce, on average, a *smaller *change in the *E_l-h _*quantity. These averages are shown as thick dashed lines and have a difference of 30 meV, which is on the order of the 14 meV difference between average changes induced by 0.1 and 0.5 eV bombardments.

### Comparison with classical molecular dynamics

In the classical molecular dynamics approach, the physical accuracy of the simulation is determined mainly by the quality of the interatomic potentials. Like its predecessor, the REBO potential, AIREBO is a member of the classical bond-order family of potentials [20,21] of the Tersoff-Brenner type, which provides a good description of the covalent bonds for nonpolar systems. The REBO potential is short ranged (< 2 Å) and, therefore, considerably less costly to use in computation but might not be suitable for collisions where long-range interactions are important, or for describing the coupling of adjacent graphene planes. REBO is also known for its poor treatment of conjugated couplings [20]. The AIREBO contains improved descriptions of the torsional and long-range van der Waals interactions (< 11 Å) as well as improved bonding interactions. The ability to use a classical (if reactive) molecular dynamics approach for the bombardment problem is highly desirable since these approaches are orders of magnitude computationally cheaper than even SCC-DFTB.

**Figure 10:**
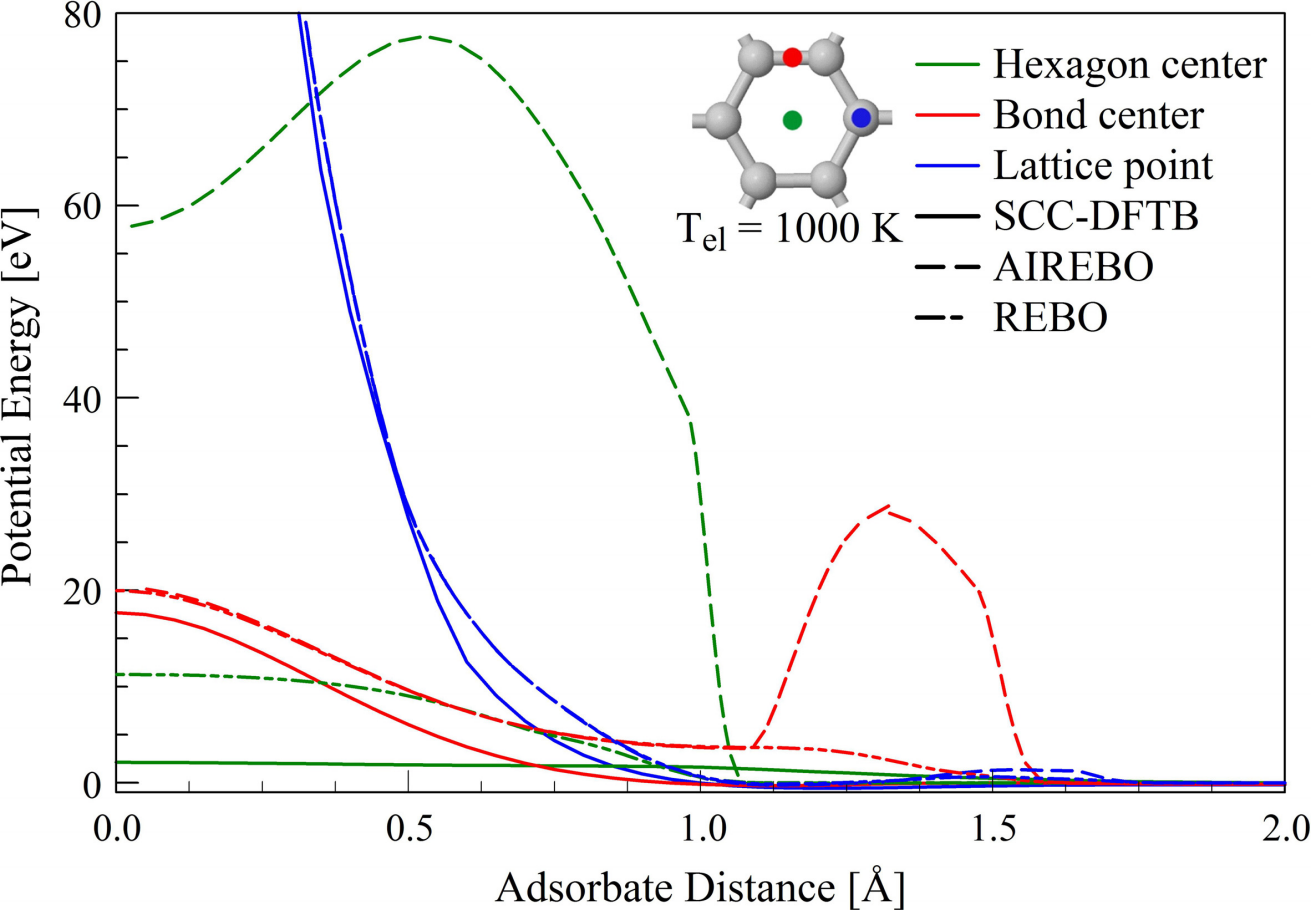
**Potential energy of the H-graphene interaction at canonical points in the lattice**. As calculated in DFTB (solid), AIREBO (single dash), and REBO (double dash).

Figure 10 shows a comparison of the refitted [22] AIREBO and REBO H-graphene potentials with that of SCC-DFTB shown in Figure 2. The CMD potentials were calculated using a 480-atom graphene *cluster*, i.e., no periodic boundary conditions. All three potentials display a well near 1.2 Å for incidence upon a lattice carbon, and the subsequent repulsive barriers agree well as they are all fit to ZBL [22]. However, REBO and AIREBO predict 0.5 and 1.0 eV barriers, respectively, before the potential wells. As these barriers are not present in the SCC-DFTB potential, it is expected that REBO and AIREBO result in different dynamics at low-energy bombardment. The dissimilarities are even more distinct for the other positions in the lattice. AIREBO predicts a potential barrier of over 20 eV, peaking at about 1.35 Å, for incidence on the C-C bond center. Neither DFTB nor REBO agree with this barrier, which is produced by the long-range Lennard-Jones terms in AIREBO, since the AIREBO and REBO results are indistinguishable at distances less than 1 Å. Another peak of 20 eV height is found at *z = *0 for AIREBO and REBO, only 2 eV higher than the corresponding SCC-DFTB curve. REBO is consistently 2 to 5 eV more repulsive than DFTB, but qualitatively very similar. The most distinctive difference between these three potentials is their treatment of the graphene π-orbitals. Clearly, the AIREBO Lennard-Jones interactions coming from the six adjacent carbons produce a potential barrier at the graphene hexagon center that is more than 60 eV (525%) higher than the potential in its predecessor, which is in turn roughly 10 eV (380%) higher than DFTB. The REBO potentials clearly agree much more with the DFTB calculations than those of AIREBO, which indicates that the Lennard-Jones interactions which produce the observed potential barriers likely overestimate the hydrogen-graphene interaction.

**Figure 11:**
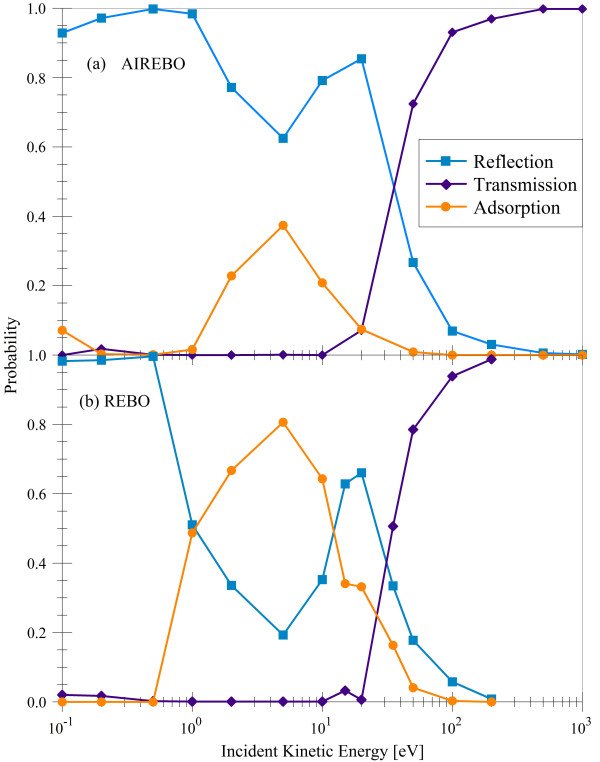
**Probabilities of reflection, transmission, and adsorption as calculated by AIREBO and REBO **[5,30]. The presence of potential barriers before potential wells (see Figure 10) results primarily in reflection at low incident energies.

Ito et al. [6,30], Nakamura et al. [5], and Saito et al. [33] have done a comprehensive study of the response of a single graphene sheet (reflection, transmission, and absorption) to the impact of hydrogen atoms and its isotopes in an energy range below 200 eV. They used classical molecular dynamics simulations with the short-range (< 2 Å) modified Brenner (REBO) potential. Unfortunately, the distribution of barriers and wells is not clear for their modification of the potential; however, their calculation of the reflection, transmission, and adsorption probabilities upon normal impact shows good qualitative agreement with our AIREBO calculations, as illustrated in Figure 11. Classical MD AIREBO and REBO results are significantly different than the quantum-classical SCC-DFTB results mainly due to the presence of the potential barriers observed in Figure 10. The AIREBO and REBO graphene potentials are more repulsive than those of SCC-DFTB, which results in a 15 eV higher threshold for transmission to occur. However, all methods converge to 100% transmission at high energies, as has been observed in previous studies [8]. While REBO shows a much higher peak in adsorption probability, the presence of the aforementioned barriers in both potentials result in a dominance of reflection at low energies, inconsistent with the results of SCC-DFTB presented above.

**Figure 12:**
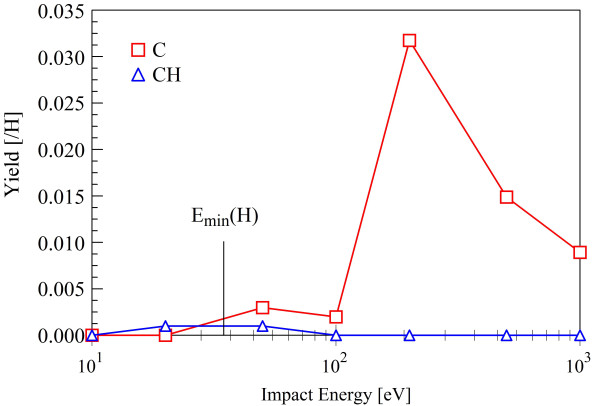
**Sputtering yields of C and CH as determined by AIREBO simulations**.

Another result of the increased repulsiveness of AIREBO is the occurrence of physical carbon sputtering upon impact of hydrogen. Figure 12 shows the sputtering yield as a function of incident kinetic energy for AIREBO calculations. If *E_d _*is a carbon atom displacement energy from the rapheme, then the kinetic energy of the impact atom in the head-on binary collision is $E_{\text{min}}^{\text{sput}}=E_{d}{\left(m_{i}+m_{c}\right)}^{2}/\left(4m_{i}m_{c}\right)$ The known energy for displacing one atom from a pristine rapheme is 22.2 eV, which yields $E_{\text{min}}^{\text{sput}}$(H) = 78.2 eV. Consistently, the sputtering yields in Figure 12 for all sputtered species are zero at 20 eV and start rising from 50 eV impact energy (where the sputtering yield is approximately 0.002). After a peak of 0.0325 at about 200 eV, they then decrease with higher incident energy. Chemical sputtering, i.e., production of CH, which is a second-order process (breaking of a carbon bond followed by capture of H by the carbon atom) here, is quite improbable, and its yield stays well below 0.005. We note again that no sputtering, physical or chemical, is measured in the SCC-DFTB simulations in the considered range of impact H energies (< 200 eV).

**Figure 13:**
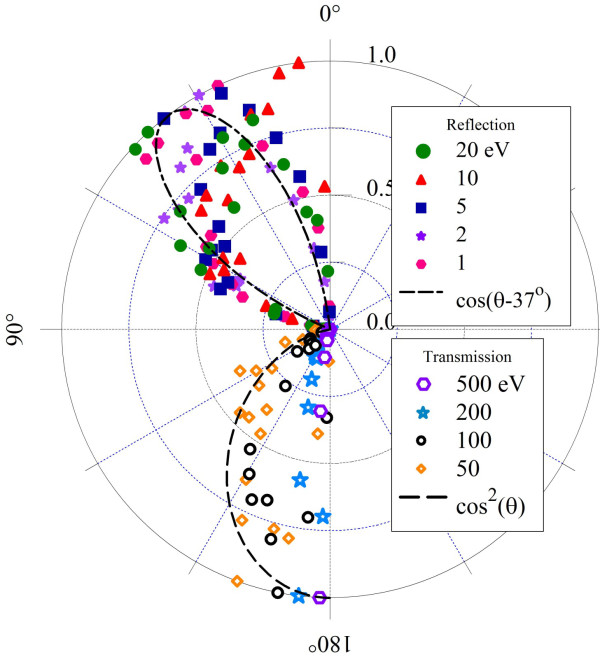
**Angular distributions of reflected (*θ *< 90°) and transmitted (*θ *> 90°) hydrogen atoms**. As calculated in AIREBO CMD simulations.

Lastly, the CMD calculations result in larger angular scattering effects than SCC-DFTB, as can be seen in Figure 13. The reason for the markedly different distribution is again in the potential barriers that arise from the Lennard-Jones interactions. The stronger interaction produces a more significant change in the incident hydrogen *x- *or *y-*momentum, resulting in a cos(*θ-θo*) distribution with maximum at roughly 37°. Transmitting atoms also interact with the potential barriers on the opposite side of the surface, which are responsible for deflecting these atoms and producing the much wider distribution than observed in the SCC-DFTB calculations.

## Conclusions

Understanding the effects of irradiation is paramount in developing graphene-based nanosensors and nanoelectronics. Thus, in this work, simulations of single-layer graphene bombarded by hydrogen atoms for a wide range of incident energies were carried out using quantum-classical molecular dynamics based on the self-consistent-charge-density functional tight binding method for treatment of the electron dynamics, combined with classical dynamics of the nuclei. The effects of this bombardment on the graphene sheet and the scattered particle distributions were analyzed in terms of reflection, transmission, and adsorption probabilities and angular distributions. Particularly significant effects of adsorption on the graphene *E_l-h _*quantity, analogous to the HOMO-LUMO gap in clusters, were investigated, predicting a notable change of the graphene electrical conductivity for even one H-atom chemisorbed. Adsorption was found to be the dominant process below 1 eV, with transmission dominating above 20 eV and reflection dominating at the intermediate energies. Reflection was found to have a more significant scattering effect than transmission.

A comparison between results of the SCC-DFTB simulations and classical MD simulations employing the AIREBO potential was made, showing a significant difference in the calculated probabilities and chemistry, mainly caused by differences in the multibody potentials. The AIREBO H-graphene potential overestimates (in comparison to SCC-DFTB) the interaction at the hexagon center (π-orbital) and C-C bond center (σ-orbital) lattice positions. A comparison of REBO and AIREBO showed that the overestimate is a result of the Lennard-Jones terms in AIREBO. The effect of this added repulsiveness permeated all of the dynamics, producing wider scattering, a much smaller adsorption probability, and nonzero sputtering yields. Refitting of these terms may significantly improve the accuracy of AIREBO.

Changes in the graphene *E_l-h _*quantity, qualitatively associated to the H-L gap and electric conductance of graphene, were found to depend on incident atom energy. Using an averaging time of 32 fs, in addition to averaging over all adsorbed trajectories, the adsorption effect on the *E_l-h _*differed by roughly 10 meV between incident energies. By virtue of higher vibrational energy, larger incident kinetic energies are found to have a smaller effect on the band gap, as shown in Figure 9. Further characterization of the *E_l-h _*changes and/or adsorbed vibrational modes could support the application of graphene in a hypersensitive slow single-particle detector in agreement with the sensitivity to a single biomolecule being coupled to a graphene sheet [34]. This hypersensitivity of the *E_l-h _*quantity to hydrogen adsorption indicates that the functionality of graphene-based nanoelectronics could be adversely affected by the irradiation by light, chemically reactive species.

## Competing interests

The authors declare that they have no competing interests.

## Authors' contributions

RCE carried out computations, and analyzed results with PSK and JD. RCE and PSK prepared the manuscript, which was finalized together with JD, JJ, and PRCK. JJ provided help in using DFTB. All authors read and approved the final manuscript.

## References

[B1] Dimitrakakis G Tylianakis E Froudakis G Pillared graphene: a new 3-D innovative network nanostructure for enhanced hydrogen storage Nano Lett 2008 8 10 3166 3170 10.1021/nl801417w 18800853

[B2] Deretzis I Fiori G Iannaccone G Piccitto G La Magna A Quantum transport modeling of defected graphene nanoribbons Physica E in press doi: 10.1016/j.physe.2010.06.024

[B3] Gorjizadeh N Kawazoe Y Chemical functionalization of graphene nanoribbons J Nanomaterials 2010 2010 1 7

[B4] Schedin F Geim AK Morozov SV Hill EW Blake P Katsnelson MI Novoselov KS Detection of individual gas molecules adsorbed on graphene Nat Mater 2007 6 652 655 10.1038/nmat1967 17660825

[B5] Nakamura H Takayama A Ito A Molecular dynamics simulation of hydrogen isotope injection into graphene Contrib Plasma Phys 2008 48 265 269 10.1002/ctpp.200810046

[B6] Ito A Nakamura H Molecular dynamics simulation of bombardment of hydrogen atoms on graphite surface Commun Comput Phys 2008 4 592 610

[B7] Krasheninnikov A Nordlund K Ion and electron irradiation-induced effects in nanostructured materials J Appl Phys 2010 107 071301 10.1063/1.3318261

[B8] Lehtinen O Kotakoski J Krasheninnikov AV Tolvanen A Nordlund K Keinonen J Effects of ion bombardment on a two-dimensional target: atomistic simulations of graphene irradiation Phys Rev B 2010 81 153401

[B9] Wakabayashi K Takane Y Yamamoto M Sigrist M Electronic transport properties of graphene nanoribbons N J Phys 2009 11 095016 10.1088/1367-2630/11/9/095016

[B10] Porezag D Frauenheim T Kohler T Seifert G Kaschner R Construction of tight-binding-like potentials on the basis of density-functional theory: application to carbon Phys Rev B 1995 51 12947 12957 10.1103/PhysRevB.51.12947 9978089

[B11] Elstner M Porezag D Jungnickel G Elsner J Haugk M Frauenheim T Suhai S Seifert G Self-consistent-charge density-functional tight-binding method for simulations of complex materials properties Phys Rev B 1998 58 7260 7268 10.1103/PhysRevB.58.7260

[B12] Oliviera AF Seifert G Heine T Duarte HA Density-functional based tight-binding: an approximate DFT method J Braz Chem Soc 2009 20 7 1193 1205 10.1590/S0103-50532009000700002

[B13] Rauls E Elsner J Gutierrez R Frauenheim T Stoichiometric and non-stoichiometric (1010) and (1120) surfaces in 2H-SiC: a theoretical study Solid State Comm 1999 111 8 459 464 10.1016/S0038-1098(99)00137-4

[B14] Ziegler JF Biersack JP Littmark U The Stopping and Range of Ions in Matter 1985 New York: Pergamon

[B15] Androitis AN Menon M Srivastava D Froudakis G Extreme hydrogen sensitivity of the transport properties of single-wall carbon-nanotube capsules Phys Rev B 2001 64 193401

[B16] Berashevich J Chakraborty T Tunable band gap and magnetic ordering by adsorption of molecules on graphene Phys Rev B 2009 80 3 2 45

[B17] Gao H Wan L Zhao J Ding F Lu J Band gap tuning of hydrogenated graphene: H coverage and configuration dependence J Phys Chem C 2011 115 8 3236 3242 10.1021/jp1094454

[B18] Elias DC Nair RR Mohiuddin TMG Morozov SV Blake P Halsall MP Ferrari AC Boukhvalov DW Katsnelson MI Geim AK Novoselov KS Control of graphene's properties by reversible hydrogenation Science 2009 323 610 630 10.1126/science.1167130 19179524

[B19] McKay H Wales DJ Jenkins SJ Verges JA de Andres PL Hydrogen on graphene under stress: molecular dissociation and gap opening Phys Rev B 2010 81 07542

[B20] Brenner DW Shenderova OA Harrison JA Stuart SJ Ni B Sinnott SB A second-generation reactive empirical bond order (REBO) potential energy expression for hydrocarbons J Phys Condens Matter 2002 14 783 802 10.1088/0953-8984/14/4/312

[B21] Stuart SJ Tutein AB Harrison JA A reactive potential for hydrocarbons with intermolecular interactions J Chem Phys 2000 112 6472 6486 10.1063/1.481208

[B22] Kent PRC Dadras J Krstic PS Improved hydrocarbon potentials for sputtering studies J Nucl Mater 2011 415 S183 S186 10.1016/j.jnucmat.2010.08.051

[B23] Yang M Nurbawono A Zhang C Feng YP Ariando Two-dimensional graphene superlattice made with partial hydrogenation App Phys Lett 2010 96 193115 10.1063/1.3425664

[B24] Vosko SH Wilk LH Nussair M Accurate spin-dependent electron liquid correlation energies for local spin density calculations: a critical analysis Can J Phys 1980 58 8 1200 1211 10.1139/p80-159

[B25] Jeloaica L Sidis V DFT investigation of the adsorption of atomic hydrogen on a cluster-model graphite surface Chem Phys Lett 1999 300 157 162 10.1016/S0009-2614(98)01337-2

[B26] Jakowski J Irle S Mrokuma K Collision-induced fusion of two C_60 _fullerenes: quantum chemical molecular dynamics simulations Phys Rev B 2010 82 12 125443

[B27] Zheng G Irle S Morokuma K Performance of the DFTB method in comparison to DFT and semiempirical methods for geometries and energies of C20-C86 fullerene isomers Chem Phys Lett 2005 412 210 216 10.1016/j.cplett.2005.06.105

[B28] Zheng G Lundberg M Jakowski J Morokuma K Implementation and benchmark tests of the DFTB method and its application to the ONIOM method Int J Quantum Chem 2009 109 1841 1854 10.1002/qua.22002

[B29] Elstner M The SCC-DFTB method and its application to biological systems Theor Chem Acc 2005 116 316 325

[B30] Ito A Nakamura H Takayama A Molecular dynamics simulation of the chemical interaction between hydrogen atom and graphene J Phys Society Japan 2008 77 114602 10.1143/JPSJ.77.114602

[B31] Zhou J Wu MM Zhou X Sun Q Tuning electronic and magnetic properties of graphene by surface modification App Phys Lett 2009 95 10 103108 10.1063/1.3225154

[B32] Klintenberg M Lebegue S Katsnelson MI Eriksson O Phys Rev B 2010 81 8 085433

[B33] Saito S Ito A Nakamura H Incident angle dependence of reactions between graphene and hydrogen atom by molecular dynamics simulation Annual Report of National Institute for Fusion Science 2010 958

[B34] Nelson T Zhang B Prezhdo OV Detection of nucleic acids with graphene nanopores: ab initio characterization of a novel sequencing device Nano Lett 2010 10 3237 3242 10.1021/nl9035934 20722409

